# Normalisation of cerebrospinal fluid biomarkers parallels improvement of neurological symptoms following HAART in HIV dementia – case report

**DOI:** 10.1186/1471-2334-6-141

**Published:** 2006-09-15

**Authors:** Lars-Magnus Andersson, Lars Hagberg, Lars Rosengren, Dietmar Fuchs, Kaj Blennow, Magnus Gisslén

**Affiliations:** 1 Department of Infectious Diseases, Sahlgrenska University Hospital, Göteborg, Sweden; 2 Department of Neurology, Sahlgrenska University Hospital, Göteborg, Sweden; 3 Division of Biological Chemistry, Biocentre, Ludwig-Boltzman Institute for AIDS Research, Medical University of Innsbruck, Innsbruck, Austria; 4 Department of Psychiatry and Neurochemistry, Institute of Clinical Neuroscience, Sahlgrenska University Hospital, Göteborg, Sweden

## Abstract

**Background:**

Since the introduction of HAART the incidence of HIV dementia has declined and HAART seems to improve neurocognitive function in patients with HIV dementia. Currently, HIV dementia develops mainly in patients without effective treatment, though it has also been described in patients on HAART and milder HIV-associated neuropsychological impairment is still frequent among HIV-1 infected patients regardless of HAART. Elevated cerebrospinal fluid (CSF) levels of markers of neural injury and immune activation have been found in HIV dementia, but neither of those, nor CSF HIV-1 RNA levels have been proven useful as diagnostic or prognostic pseudomarkers in HIV dementia.

**Case presentation:**

We report a case of HIV dementia (MSK stage 3) in a 57 year old antiretroviral naïve man who was introduced on zidovudine, lamivudine and ritonavir boosted indinavir, and followed with consecutive lumbar punctures before and after two and 15 months after initiation of HAART. Improvement of neurocognitive function was paralleled by normalisation of CSF neural markers (NFL, Tau and GFAP) levels and a decline in CSF and serum neopterin and CSF and plasma HIV-1 RNA levels.

**Conclusion:**

The value of these CSF markers as prognostic pseudomarkers of the effect of HAART on neurocognitive impairment in HIV dementia ought to be evaluated in longitudinal studies.

## Background

Neurological complications are common in HIV-1 infection and HIV-1 can be detected in cerebrospinal fluid (CSF) or brain tissue in all stages of infection[1,2]. HIV dementia, also termed HIV associated dementia or AIDS dementia complex, is a subcortical dementia which is characterised by a combination of cognitive/behavioural and motor dysfunction. The diagnosis is based on clinical criteria and the absence of other diseases that may explain the condition and requires a thorough clinical examination along with radiological imaging of the brain and CSF analyses. About half of patients with HIV dementia have neuropathological findings consistent with HIV-1 encephalitis, which is characterised by multinucleated giant cells, microglial nodules, perivascular inflammation, astrocytosis, myelin pallor, and neuronal loss[3,4]. There seem to be regional differences in neuropathology and viral load. Subcortical structures such as basal ganglia, especially nucleus caudatus, and the hippocampus appear to be most vulnerable[3,5].

The exact mechanisms of neuronal injury in HIV-1 infection are not known. However, as cells of macrophage/microglial-lineage are the only productively infected cells in the central nervous system (CNS) an indirect neurotoxic effect has been suggested [6-8]. Intrathecal immunoactivation is probably also part of the neuropathogenicity and neurodegeneration is most often accompanied by increased immunostimulation [9-11].

Neopterin is a marker of cellular immune activation and is secreted by monocyte-derived macrophages when stimulated by interferon gamma. High CSF neopterin levels have been found in HIV-1 infected patients with HIV dementia and CNS opportunistic disease[12,13]. Neurofilament is a major structural component of myelinated axons. The light chain of the neurofilament protein (NFL) can be used as a marker of CNS injury. Elevated CSF levels of NFL have been found in cerebrovascular disease, multiple sclerosis, herpes simplex encephalitis, amyotrophic lateral sclerosis, and AIDS[14,15]. Protein tau (Tau) is a microtubuli associated protein that promotes microtubuli assembly and stability. A hyperphosphorylated form of Tau (Paired helical filaments (PHF)-Tau) is found in brain tissue from patients with Alzheimer's disease. Elevated levels of normal and PHF-Tau have been found in Alzheimer's disease, vascular dementia, HIV dementia, and HIV-1 infected patients with CNS opportunistic disease[9,16]. The glial fibrillary acidic protein (GFAP) is the structural protein of the glial intermediate filament, which constitutes the morphological basis of astrogliosis. Increased CSF levels of GFAP have been demonstrated in patients with Alzheimer's disease, vascular dementia, multiple sclerosis, and AIDS[17,18]. Elevated CSF levels of S-100 protein (S100), a marker of astroglial cell destruction, and neuron specific enolase (NSE), a marker of neural injury, have been found in herpes simplex encephalitis[19]. Blood-brain barrier (BBB) impairment, as measured by the albumin ratio, has been found in HIV-1 infected patients in various stages of disease and the proportion of HIV-1 infected individuals with an elevated albumin ratio seems to increase with more advanced disease [20-22]. In fact, some groups have reported impaired BBB integrity in 100% of patients with HIV-encephalitis[23,24]. IgG index is used as a measurement of intrathecal antibody production. High IgG index values have been found in HIV-1 infected patients with neurological complications[25]. High CSF HIV-1 RNA levels have been found in patients with HIV dementia but neither CSF HIV-1 RNA levels nor markers of immune activation, neural injury or BBB dysfunction have been proven useful as prognostic or diagnostic markers in HIV dementia [26-29].

CSF and serum neopterin were analysed by radio-immunoassay (Henningtest neopterin, Brahms, Berlin, Germany), CSF NFL, Tau, and GFAP by previously described enzyme linked immunoassays[14,16,17], and CSF and plasma HIV-1 RNA by quantitative PCR (Roche UltraSensitive HIV Monitor and Amplicor HIV Monitor version 1.5, Hoffman La-Roche, Basel, Switzerland). Samples with HIV-1 RNA levels exceeding the upper limit of the dynamic range of the UltraSensitive HIV Monitor test (>75000 HIV-1 RNA copies/ml) were reanalysed with the Amplicor HIV Monitor test (dynamic range 400–750000 HIV-1 RNA copies/ml).

Since the introduction HAART the incidence of HIV dementia has declined[30,31]. Several groups have reported improved neurocognitive function after initiation of HAART, while others have found continued decline in neurologic function in a subset of individuals despite good virological control [32-34].

We report a case of HIV dementia in a patient who was followed with repeated CSF sampling before and after initiation of HAART and where neurocognitive improvement was paralleled by declining levels of CSF markers of neural injury.

## Case presentation

**Figure 1:**
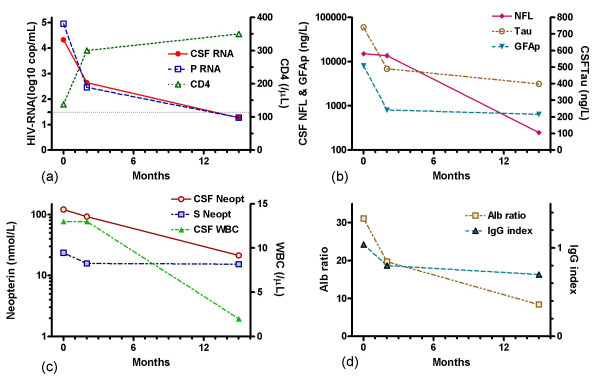
Cerebrospinal fluid (CSF) and plasma HIV-1 RNA (log10 copies/ml), and CD4 cell count (cells/μl) (a); CSF NFL (* <250 ng/L), CSF Tau (* <400 ng/L), and CSF GFAP (*<750 ng/L) (b); CSF neopterin (* <4.2 nmol/L) and serum neopterin (* <8.8 nmol/L), and CSF mononuclear cell count (cells/μL) (c); albumin ratio (* <10.2) and IgG index (*<0.63) (d); before, and after two and 15 months after initiation of HAART. *Upper limit of normal.

A 57 year old previously healthy man was admitted to the Department of Infectious Diseases, Sahlgrenska University Hospital, Göteborg, Sweden in January 2000. He presented with a three month history of increasing apathy, gait difficulties and memory dysfunction. On examination he showed normal vital signs. Neurological examination revealed ataxic gait, memory impairment, reduced attention and slow verbal response. A HIV-1 antibody test was positive and his CD4-cell count was 50 ×10e6 cells/l. A brain MRI scan revealed no intracranial pathology except for areas of increased signal changes in T2 MRI sequences in periventricular white matter. Following MRI scan a lumbar puncture and additional blood tests were performed. Routine laboratory tests were normal except for an elevated sedimentation rate (76 mm/h). Tests and cultures in CSF and blood for bacterial, mycobacterial, fungal and protozoal infections, including PCR tests for JC-virus, cytomegalovirus, herpes simplex virus, varicella zoster virus, Epstein-Barr virus, and toxoplasmosis in CSF, antigen test for cryptococci in CSF, and antibody test for syphilis in CSF and blood, were negative. CSF PCR tests for diagnostic purposes were run as soon as possible after sampling. CSF cytology was normal. Thus, brain MRI scan and CSF assessment showed no evidence of other infections or malignancies. CSF and plasma HIV-1 RNA levels, CSF cell counts, and CD4 cell counts are presented in figure 1. After additional neurological and psychiatric tests, performed by an experienced neurologist, a diagnosis of HIV dementia (MSK stage 3)[35,36] was made. The patient was introduced on zidovudine 300 mg BID, lamivudine 150 mg BID, indinavir 800 mg BID and ritonavir 100 mg BID p.o. in January 2000 and was then followed with physical and neurological examination and CSF assessment after 2 and 15 months. He showed a gradual neurocognitive improvement that was paralleled by a decline in plasma and CSF HIV-1 RNA levels as well as levels of CSF NFL, Tau, GFAP, neopterin, TAU, albumin ratio, and IgG index (figure 1). CSF levels of S-100 protein and NSE were below the upper limit of normal before treatment initiation. Neurological examination after 15 months of HAART showed no remaining neurocognitive impairment and the patient had resumed all activities of daily living, as well as full time work.

## Discussion

Although the incidence of HIV dementia has declined since the introduction of HAART[30,31], the effect of HAART on HIV dementia has not been as thoroughly evaluated. Also, the long term effects of HAART on neurological impairment in HIV-1 infection are not well known and there have been reports of continued neurological impairment in spite of virological control in some patients with HIV dementia[33]. We have previously found elevated CSF neopterin levels after 2 years of continuous HAART in neuroasymptomatic HIV-1 infected patients[37] and recently reported increasing CSF levels of NFL in a subset of neuroasymptomatic HIV-1 infected patients after discontinuation of HAART[38].

In the case reported here, complete neurocognitive recovery was paralleled by normalisation of CSF-levels of NFL and HIV-1 RNA, indicating that the process of axonal damage might have been halted by the CSF penetrating combination of zidovudine, lamivudine, ritonavir and indinavir. It seemed as if CSF NFL decrease was slow compared to the decrease in CSF Tau and GFAP levels. Slow CSF NFL normalisation has been reported in herpes simplex type 1 encephalitis, focal brain ischemia, and multiple sclerosis[19,39]. Whether this reflects slow metabolic degradation and a long half-life of NFL in CSF or different immunopathological pathways that lag behind the decrease in HIV-1 replication in CNS is not clear. In our patient CSF WBC and neopterin followed the same pattern as CSF NFL indicating a correlation with intrathecal immune activation. The decline in albumin ratio and IgG index values was in concordance with previous reports on effects of HAART on BBB integrity and intrathecal antibody production[40].

Before the introduction of HAART, two-thirds of patients with HIV dementia followed a rapid course with fast deterioration and death while the other third had a slower clinical course with prolonged survival[41]. In recent reports it seems that some patients with HIV dementia experience rapid neurocognitive improvement while others have a less pronounced or no effect after starting HAART [32-34]. If this is explained by different mechanisms of neural injury or if some patients develop irreversible brain damage prior to treatment is not known.

## Conclusion

Rapid decline in CSF markers of axonal damage, as described here, indicates that CSF NFL, or a combination of markers of neural injury, might be of value as an indicator of the effect of HAART on neurocognitive impairment in patients with HIV dementia and merits evaluation as a prognostic pseudomarker in treatment of HIV dementia in longitudinal studies.

## Competing interests

The author(s) declare that they have no competing interests.

## Authors' contributions

LMA, MG and LH contributed to data interpretation and writing of the paper. KB and LR contributed to analysis of CSF markers of neuronal damage and writing of the paper. DF contributed to CSF and serum neopterin analysis and writing of the paper.

## Pre-publication history

The pre-publication history for this paper can be accessed here:


